# Professional Identity of 0.24 Million Medical Students in China Before and During the COVID-19 Pandemic: Three Waves of National Cross-Sectional Studies

**DOI:** 10.3389/fpubh.2022.868914

**Published:** 2022-03-25

**Authors:** Chen Yu, Qiao Liu, Weimin Wang, Ana Xie, Jue Liu

**Affiliations:** ^1^National Center for Health Professions Education Development, Peking University, Beijing, China; ^2^Department of Medical Education, Peking University, Beijing, China; ^3^Department of Epidemiology and Biostatistics, School of Public Health, Peking University, Beijing, China; ^4^Peking University Health Science Center, Beijing, China; ^5^Department of Global Health and Development, Peking University, Beijing, China

**Keywords:** professional identity, professional identity formation, medical students, COVID-19, national cross-sectional survey

## Abstract

**Background:**

Professional identity (PI) influences the doctor's thoughts and behaviors. Thus, PI formation (PIF) plays an important role in medical students' education. Major changes to the learning environment could impact PIF, but the influence of the novel coronavirus disease 2019 (COVID-19) pandemic on medical students' PI had confusing conclusions in previous studies. We aimed to compare PI of medical students by using the data from three waves of national cross-sectional surveys conducted in China in 2019, 2020, and 2021, and to examine factors that influence PIF.

**Method:**

We used data from the China Medical Student Survey (CMSS) which has conducted three national cross-sectional surveys. From 2019 to 2021, CMCC retrieved data on PI from a nationally representative sample of medical students from 33, 121, and 123 colleges, respectively. We analyzed the data using Chi-square test, analysis of variance, and multivariable logistic regression according to sociodemographic characteristics, pre-university experience, college characteristics, and college experience.

**Results:**

A total of 244,040 medical students in China participated in the surveys. The overall score of PI increased from 3.80 in 2019 to 3.85 in 2021. Medical students with family medical background, high intrinsic and extrinsic motivation of major selection, teachers' positive role model, and high personal comprehensive quality ranking were more likely to have higher PI (all *p* < 0.05). The more attention students paid to the COVID-19 pandemic, the higher PI they would have (aOR 1.93, 95% CI 1.67–2.24 for more attention; aOR 2.31, 95% CI 2.00–2.68 for the most attention). However, parents' participation on the front lines of COVID-19 pandemic negatively influenced the PI of medical students (aOR 0.72, 95% CI 0.57–0.93).

**Conclusions:**

PI of medical students increased during the COVID-19 pandemic. The impact of the pandemic on PI was complex. To improve the PI of medical students, the education sector, health sector and the society need to make concerted efforts.

## Introduction

Professional identity (PI) refers to people's professional perception of themselves based on their beliefs and values. It leads the way they think, behave, and interact with professional and social norms (1). PIs influence the doctor's thoughts and behaviors, hence, PI formation (PIF)was suggested to be a major focus of medical education (2, 3). PIF is now recognized as crucial to developing doctors who can deliver high-quality care (4). Those who constructed a PI that aligns with the needs and values of the general practice environment have shown more satisfaction and emotional well-being in their roles (5). Newly graduated doctors are required to perform professionally, while there is little time for medical students to transit from student to doctor. Therefore, a well-formed PI during medical education and clinical practice is of vital importance for medical students to quickly adapt to professional status and to better deal with practical challenges. Those doctors with a strong PI could not only benefit themselves, but also positively impact their patients and coworkers (1).

Personal identity formation could be impacted by major changes to the learning environment, and medical education is now in the midst of a radical change with the novel coronavirus disease 2019 (COVID-19) pandemic (6). The pandemic could alter, impede, or accelerate the process of PIF of medical students by creating additional concerns about doctors' role in providing healthcare, the functions and limitations of medical care, and individuals' vulnerability to infection and asymptomatic disease spread (7). Educators should seize the opportunity to understand the changes of medical students' PI under the pandemic and formulate targeted measures to help medical students form a better PI.

We searched PubMed to identify full-text reports that were relevant to our study aims and found that several studies had examined the influence of COVID-19 pandemic on medical students' PI, but with contrary conclusions. A cross-sectional study conducted in Zhengzhou, China that included 474 nursing students found that anxiety during the COVID-19 pandemic gave an adverse effect on the PI of nursing students (8). Another nation-wide cross-sectional study identified an increased level of PI among Chinese nursing students during the COVID-19 pandemic (9). Ardi et al. found that the socialization processes that promotes PIF might change due to the tremendous disruption brought by the pandemic (10). Notably, this study was a qualitative research, particularly because of how they assessed undergraduate medical students' adaptations and PIF during the pandemic by exploring their written reflections. These studies showed that reliable and accurate information on the PI level of medical students and its change during the COVID-19 pandemic is urgently needed.

In the present study, we aimed to compare PI of medical students by using the data from three waves of national cross-sectional surveys in China conducted in 2019, 2020 and 2021, which represent students' PI before and during the COVID-19 pandemic (based on the COVID-19 prevalence in China).

## Methods

### Study Design, Participants, and Sampling

Our data was collected by the China Medical Student Survey (CMSS). CMSS is a nationwide large-scale survey project for medical undergraduates in China which was jointly initiated by the Peking University National Center for Health Professions Education Development and the Association for Health Professions Education Research in China (AHPERC). CMSS aims to understand the training process and growth experience of medical students from the perspective of students themselves, echoing the medical education certification concept of “student-centered” and “result-oriented” learning and further improve the quality assurance system of medical education. It then provides data support of policy advice and decision-making for the reform and development of medical education in China.

The China Medical Student Survey was officially launched in 2019 and has conducted three national cross-sectional surveys so far via Wen Juan Xing (Changsha Ranxing Information Technology Co., Ltd, Hunan, China), an online survey company. In 2019, stratified sampling was used, and medical students were separately sampled from “Double First-Class” universities and non- “Double First-Class” universities with a total of 10,031 medical students from 33 colleges involved. In 2020 and 2021, all colleges offering medical majors in China were investigated, and 30,395 medical students from 121 colleges and 219, 396 medical students from 123 colleges were, respectively, involved in each year of investigation.

### Measures

A structured self-administered online questionnaire was designed based on the Student Development Theory and College Impact Theory (11). The questionnaire included the following parts: (1) sociodemographic characteristics, (2) pre-university experience, (3) college characteristics, (4) college experience, and (5) PI. Sociodemographic characteristics included sex, nationality, family location, and parents' educational years. Pre-university experience included types of high school, ideal profession, and motivation of major selection scale. College characteristics included the college location and college types, while college experience included doctors' role model scale, clinical practice events scale, and personal comprehensive quality ranking during college studies. PI included a PI scale with 12 questions. In the survey in 2020, there were additional questions related to the COVID-19 pandemic. Variable selections were shown in Table 1.

**Table 1:** Variable selections.

**Variables**	**Description**	
**Independent variables**		
Professional identity score	Continuous	1–5
	Categorical	0: ≤ 3 1: >3
**Dependent variables**		
Survey year	Categorical	0: 2019 1: 2020 2: 2021
Sex	Categorical	0: male 1: female
Family medical background	Categorical	0: no 1: yes
Type of high school	Categorical	0: key high school 1: ordinary high school 2: secondary vocational colleges 3: private school
Years of education of father	Continuous	0–22
Family region	Categorical	0: Municipality directly under the Central Government/special administrative region 1: Eastern 2: Central 3: Western
College location	Categorical	0: Wuhan 1: Hubei except Wuhan 2: China except Hubei
Ideal profession related to medicine in high school	Categorical	0: no 1: yes
Intrinsic motivation score of major selection	Categorical	0: ≤3 1: >3
Extrinsic motivation score of major selection	Categorical	0: ≤3 1: >3
Positive medical behavior scores	Categorical	0: ≤3 1: >3
Negative medical behavior scores	Categorical	0: ≤3 1: >3
Positive teaching behavior scores	Categorical	0: ≤3 1: >3
Negative teaching behavior scores	Categorical	0: ≤3 1: >3
Personal events score during clinical practice	Categorical	0: ≤3 1: >3
Personal comprehensive quality ranking	Categorical	0: <10% 1: 11–25% 2: 26–50% 3: 51–75% 4: >75%
Attention to the COVID-19 pandemic	Categorical	0: general 1: more 2: most
Participation of parents or teachers on the front lines of COVID-19 pandemic	Categorical	0: neither parent nor teacher 1: only parent 2: only teacher 3: Both parent and teacher

Each scale contained several questions, and each question was answered on a five-point Likert scale (“strongly disagree,” “disagree,” “neither agree nor disagree,” “agree,” and “strongly agree”) and were assigned scores of 1, 2, 3, 4, and 5, respectively. The motivation of the major selection scale included nine questions, with five representing the intrinsic motivation and four representing the extrinsic motivation. The doctors' role model scale contained six questions, with two representing doctors' positive medical behaviors, one representing doctors' negative medical behaviors, two representing doctors' positive teaching behaviors, and one representing doctors' negative teaching behaviors. The clinical practice events scale included six questions, with two representing medical events and four representing personal events. PI consisted of four dimensions (professional cognition, professional emotion, professional behavior, and professional expectation) which were measured by three, four, three, and two questions, respectively. The content of these scales is shown in Supplementary Table 1.

The primary outcome was PI scores. The higher score refers to higher PI, and in this study, if the average scores of the 12 questions in the PI scale were more than 3, participants were then defined as having a high PI (12).

### Statistical Analysis

Descriptive statistics were performed to describe the sociodemographic characteristics and the rates of having a high PI score (>3). Analysis of variance (ANOVA) was used to compare the PI scores based on years of this study. Chi-square test was used to compare the rates of hiving a high PI score by sociodemographic characteristics, pre-university experience, college characteristics, and college experience. In each scale of the questionnaire, each variable was calculated by the average score of questions it contained. For example, the intrinsic motivation score was calculated by the scores of the five questions which represented the intrinsic motivation. If the scores of variables were over 3, participants were defined as having an agreement with the situation.

**Figure 1:**
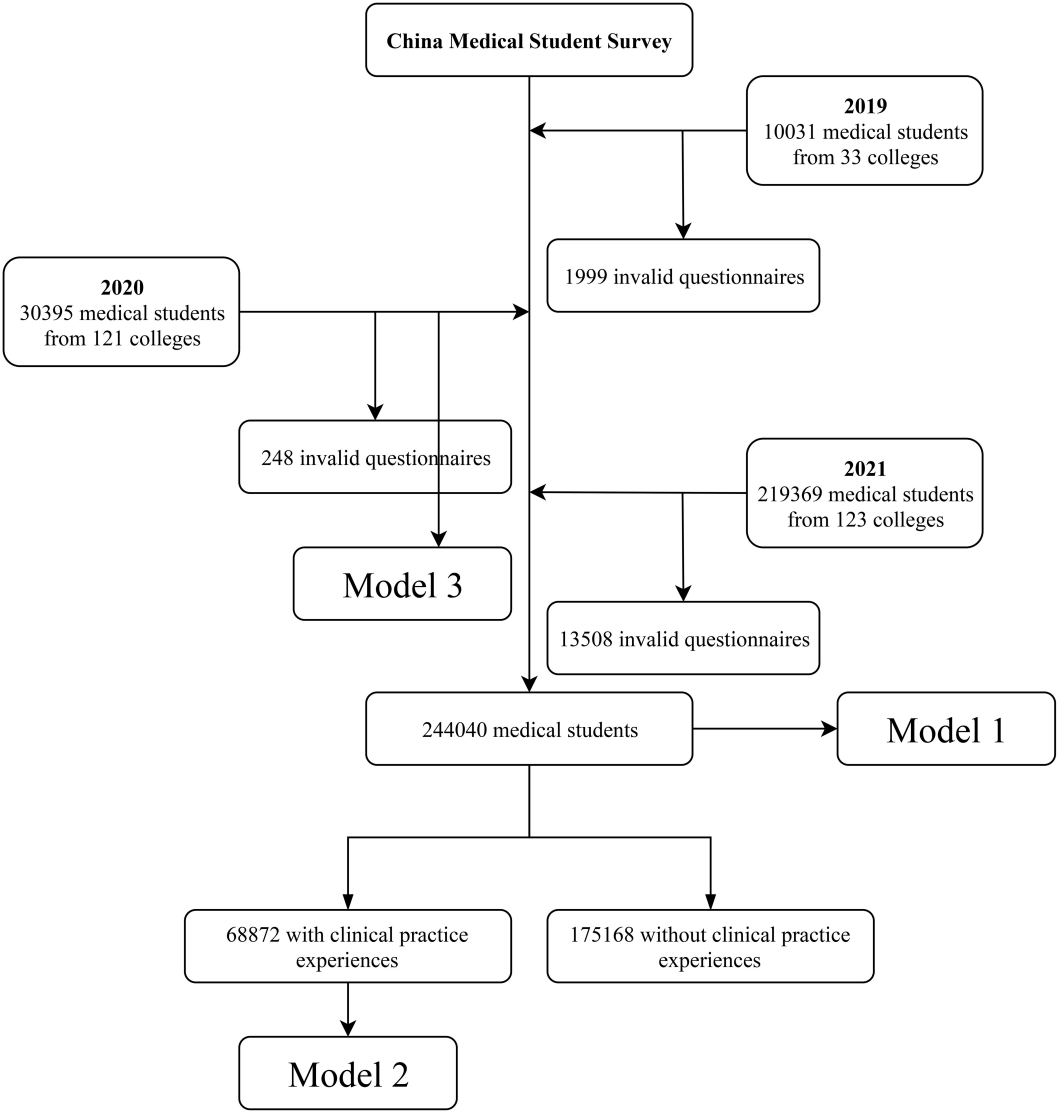
Flowchart of participants selection.

The multivariable logistic regression model was used to assess the adjusted associations of factors related to the PI which were adjusted by the survey time, sex, family location and region, family medical background, types of high school, education year of father, college type and its location, ideal profession in high school, motivation scores of major selection, doctors' medical and teaching behavior scores, personal events score during clinical practice higher, personal comprehensive quality ranking during college studies, attention to the COVID-19 pandemic, and parents' or teachers' participation on the front lines of COVID-19 pandemic. We established three logistic regression models containing different numbers of medical students with different factors according to the characteristics of participants (Figure 1). Model 1 contained all the 244,040 participants but lack the factors related to clinical practice and personal comprehensive quality rankings due to some participants have not experienced the clinical practice. On the other hand, model 2 included the factors related to clinical practice and contained 68,872 participants. Model 3 only included participants in the 2020 survey and added factors related to the COVID-19 pandemic. Adjusted odds ratios (*OR*) with 95% confident interval (*CI*) for each variable were calculated.

### Ethical Approval

The study was approved by the Institutional Review Boards at Peking University (IRB00001052-20069).

## Results

### Characteristics of Participants

A total of 259,795 medical students were involved in the three national cross-sectional surveys, with 244,040 valid questionnaires (Figure 1). Characteristics of participants were shown in Table 2. Independent medical college accounted for 67.60%. Males accounted for 53.30% of all the students involved, and the Han nationality accounted for the vast majority (88.69%). Students from urban areas were more than those from rural areas (58.16%), while only 2.94% of the students were from Hubei province. Most parents had <10 years of education and 10–20 years of education, which were relatively average, while very few had more than 20 years of education (0.65% for fathers and 0.33% for mothers). Students who had a career related to medicine in high school accounted for 52.89%. The majority of students had the intrinsic (82.61%) and extrinsic (68.24%) motivation scores of over 3 in the 3 years. During clinical practice, most of the teachers also showed to be positive role models. Medical events (2.03%) and personal events (2.44%) accounted for a small proportion in the 3 years. Differences among different PI and different years of the CMSS were in Table 2. Lastly, except for college location (*p* = 0.126), sex (*p* = 0.284), and nationality (*p* = 0.985), the differences were all statistically significant (*p* < 0.001, Table 2).

**Table 2:** Rates of professional identity score over 3 in medical students in China by demographic characteristics, and characteristics of participants in the 2019, 2020, and 2021 waves.

	**Total (*N* = 244,040) (%)**	**Professional identity scores**		**Year of surveys**		
		**>3 (*n* = 212,016) (%)**	***P* value**	**2019 *n* = 8,032 (%)**	**2020 *n* = 30,147 (%)**	**2021 *n* = 205,861 (%)**
**College location**						
Wuhan	2,877 (1.18)	2,473 (85.96)	0.13	355 (4.42)	585 (1.94)	1,937 (0.94)
Hubei except Wuhan	6,074 (2.49)	5,241 (86.29)		0 (0.00)	679 (2.25)	5,395 (2.62)
China except Hubei	235,089 (96.33)	204,302 (86.90)		7,677 (95.58)	28,883 (95.81)	198,529 (96.44)
**College type**						
Independent medical college	164,976 (67.60)	142,957 (86.65)	<0.001	3,709 (46.18)	17,525 (58.13)	143,742 (69.82)
Medical College of comprehensive university	79,064 (32.40)	69,059 (87.35)		4,323 (53.82)	12,622 (41.87)	62,119 (30.18)
**Sex**						
Male	130,065 (53.30)	113,087 (86.95)	0.28	3,590 (44.70)	12,222 (40.54)	114,253 (55.50)
Female	113,975 (46.70)	98,929 (86.80)		4,442 (55.30)	17,925 (59.46)	91,608 (44.50)
**Nationality**						
Han	216,450 (88.69)	188,045 (86.88)	0.99	7,184 (89.44)	26,939 (89.36)	182,327 (88.57)
Others	27,590 (11.31)	23,971 (86.88)		848 (10.56)	3,208 (10.64)	23,534 (11.43)
**Family location**						
Urban	141,936 (58.16)	122,848 (86.55)	<0.001	5,323 (66.27)	15,496 (51.40)	121,117 (58.83)
Rural	102,104 (41.84)	89,168 (87.33)		2,709 (33.73)	14,651 (48.60)	84,744 (41.17)
Hubei	229 (0.09)	189 (82.53)	<0.001	229 (2.85)		
Wuhan	821 (0.34)	697 (84.90)			158 (0.52)	663 (0.32)
Hubei except Wuhan	6,132 (2.51)	5,239 (85.44)			863 (2.86)	5,269 (2.56)
China except Hubei	236,858 (97.06)	205,891 (86.93)		7,803 (97.15)	29,126 (96.61)	199,929 (97.12)
**Family region**						
municipality directly under the Central Government /special administrative region	13,250 (5.43)	11,498 (86.78)	<0.001	675 (8.40)	1,358 (4.50)	11,217 (5.45)
Eastern	80,018 (32.79)	69,853 (87.30)		2,794 (34.79)	8,064 (26.75)	69,160 (33.60)
Central	66,990 (27.45)	58,201 (86.88)		2,255 (28.08)	10,249 (34.00)	54,486 (26.47)
Western	83,782 (34.33)	72,464 (86.49)		2,308 (28.74)	10,476 (34.75)	70,998 (34.49)
**Family medical background**						
Yes	71,128 (29.15)	62,660 (88.09)	<0.001	2,373 (29.54)	3,205 (10.63)	65,550 (31.84)
No	172,912 (70.85)	149,356 (86.38)		5,659 (70.46)	26,942 (89.37)	140,311 (68.16)
**Years of education of father/mother**						
~10 years	122,867 (50.35)/142,015 (58.19)	106,940 (87.04)/123,649 (87.07)	<0.001/ <0.001	3,520 (43.82)/4,180 (52.04)	17,253 (57.23)/20,062 (66.55)	102,094 (49.59)/117,773 (57.21)
10–20 years	119,648 (49.03)/101,217 (41.48)	103,799 (86.75)/87,696 (86.64)		4,417 (54.99)/3,796 (47.26)	12,766 (42.35)/10,009 (33.20)	102,465 (49.77)/87,412 (42.46)
20–years	1,525 (0.62)/808 (0.33)	1,277 (83.74)/671 (83.04)		95 (1.18)/56 (0.70)	128 (0.42)/76 (0.25)	1,302 (0.63)/676 (0.33)
**Types of high school**						
Key high school	118,630 (48.61)	103,809 (87.51)	<0.001	5,297 (65.95)	14,262 (47.31)	99,071 (48.13)
Ordinary high school	113,998 (46.71)	98,379 (86.30)		2,711 (33.75)	15,795 (52.39)	95,492 (46.39)
Secondary Vocational Colleges	481 (0.20)	353 (73.39)		24 (0.30)	90 (0.30)	367 (0.18)
Private school	10,931 (4.48)	9,475 (86.68)		0 (0.00)	0 (0.00)	10,931 (5.31)
**Was the ideal profession related to medicine in high school**						
Yes	129,080 (52.89)	117,638 (91.14)	<0.001	3,426 (42.65)	15,688 (52.04)	109,966 (53.42)
No	114,960 (47.11)	94,378 (82.10)		4,606 (57.35)	14,459 (47.96)	95,895 (46.58)
**Intrinsic motivation score of major selection**						
>3	201,611 (82.61)	187,655 (93.08)	<0.001	5,772 (71.86)	23,975 (79.53)	171,864 (83.49)
≤3	42,429 (17.39)	24,361 (57.42)		2,260 (28.14)	6,172 (20.47)	33,997 (16.51)
**Extrinsic motivation score of major selection**						
>3	166,526 (68.24)	153,268 (92.04)	<0.001	6,113 (76.11)	21,778 (72.24)	138,635 (67.34)
≤3	77,514 (31.76)	58,748 (75.79)		1,919 (23.89)	8,369 (27.76)	67,226 (32.66)
**Positive medical behavior score**						
>3	93,539 (38.33)	87,802 (93.87)	<0.001	6,460 (80.43)	23,396 (77.61)	63,683 (30.93)
≤3	30,418 (12.46)	17,465 (57.39)		1,572 (19.57)	6,751 (22.39)	22,095 (10.73)
**Negative medical behavior score**						
>3	14,803 (6.07)	13,526 (91.37)	<0.001	1,365 (16.99)	6,892 (22.86)	6,546 (3.18)
≤3	109,154 (44.73)	91,741 (85.05)		6,667 (83.01)	23,255 (77.14)	79,232 (38.49)
**Positive teaching behavior score**						
>3	88,899 (36.43)	83,459 (93.88)	<0.001	6,217 (77.40%)	22,487 (74.59%)	60,195 (29.24%)
≤3	35,058 (14.37)	21,808 (62.21)		1,815 (22.60%)	7,660 (25.41%)	25,583 (12.43%)
**Negative teaching behavior score**						
>3	18,054 (7.40)	15,970 (88.46)	<0.001	1,934 (24.08)	7,323 (24.29)	8,797 (4.27)
≤3	105,903 (43.40)	89,297 (84.32)		6,098 (75.92)	22,824 (75.71)	76,981 (37.39)
**Medical events score during clinical practice**						
>3	4,948 (2.03)	4,253 (85.95)	0.040	847 (10.55)	1,884 (6.25)	2,217 (1.08)
≤3	119,009 (48.77)	101,014 (84.88)		7,185 (89.45)	28,263 (93.75)	83,561 (40.59)
**Personal events score during clinical practice**						
>3	5,948 (2.44)	4,700 (79.02)	<0.001	831 (10.35)	2,404 (7.97)	2,713 (1.32)
≤3	118,009 (48.36)	100,567 (85.22)		7,201 (89.65)	27,743 (92.03)	83,065 (40.35)
**Personal comprehensive quality ranking**						
<10%	9,654 (3.96)	8,581 (88.89)	<0.001	1,153 (14.36)	4,432 (14.70)	4,069 (1.98)
11–25%	16,824 (6.89)	14,720 (87.49)		1,619 (20.16)	8,250 (27.37)	6,955 (3.38)
26–50%	21,968 (9.00)	18,699 (85.12)		2,525 (31.44)	9,862 (32.71)	9,581 (4.65)
51–75%	14,319 (5.87)	12,101 (84.51)		1,878 (23.38)	5,515 (18.29)	6,926 (3.36)
> 75%	6,097 (2.50)	4,648 (76.23)		857 (10.67)	2,088 (6.93)	3,152 (1.53)

**Figure 2:**
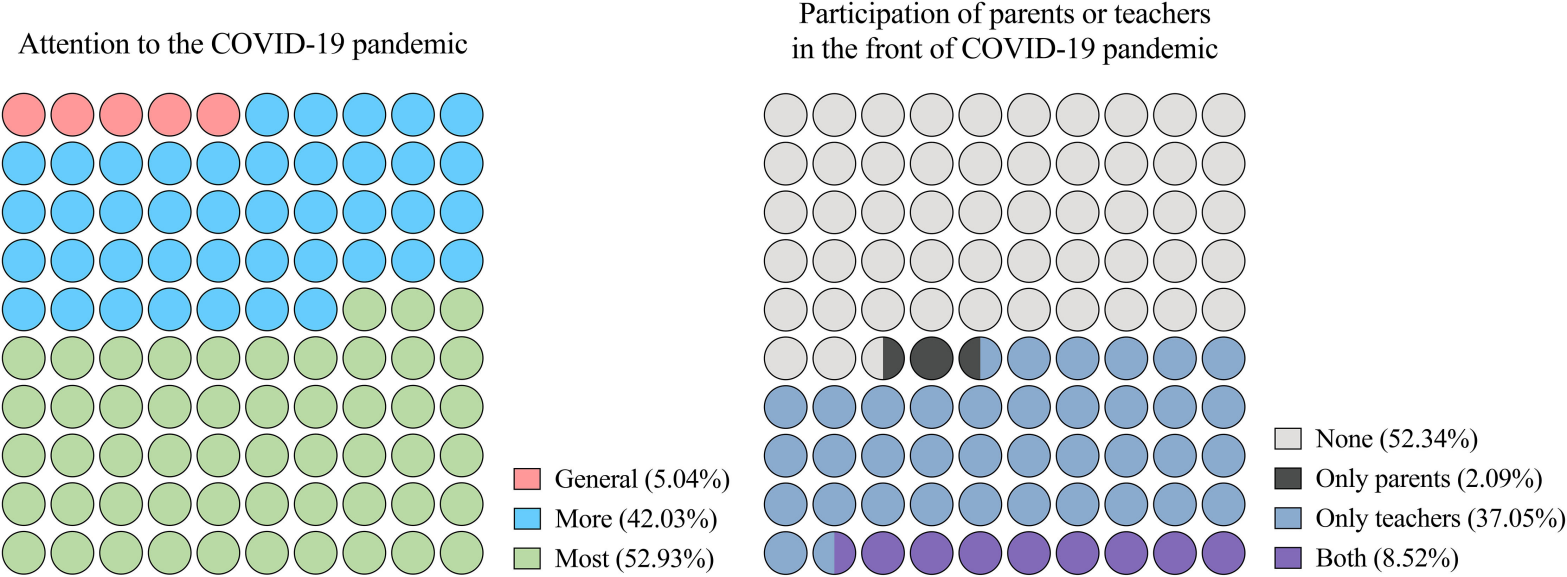
Proportion of variables related to the coronavirus disease 2019 (COVID-19) pandemic in the CMSS of 2020. COVID-19, Corona Virus Disease 2019; CMSS, China Medical Student Survey.

In 2020, only 5.04% (1,520/30,147) of the students had a general involvement and attention to the COVID-19 pandemic, while 42.03% (12,670/30,147) were more involved and therefore had more attention, and 52.93% (15,957/30,147) had the most involvement and attention to the COVID-19 pandemic compared to other participants. Fifty-two-point-thirty-four percent (15,780/30,147) of students' parents and teachers did not participate on the front lines of COVID-19 pandemic (Figure 2).

### Professional Identity of Medical Students

**Figure 3:**
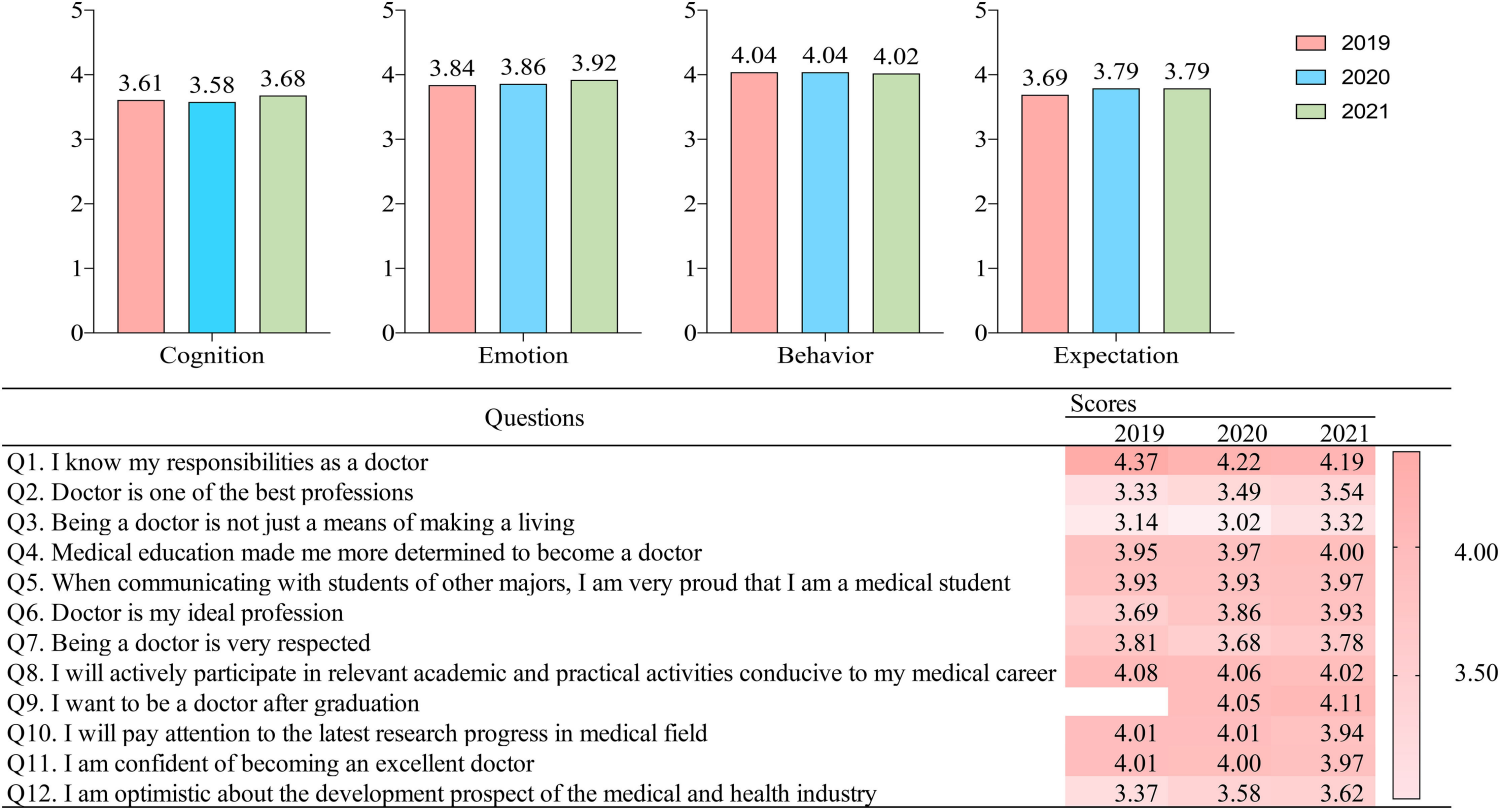
Results of professional identity scale. Professional Cognition is represented by Q1 to Q3; Professional Emotion is represented by Q4 to Q7; Professional Behavior is represented by Q8 to Q10; and Professional Expectation is represented by Q11 and Q12.

Professional identity score slightly increased from 2019 (3.80) to 2021 (3.85). ANOVA of the 3 years overall PI scores showed that the differences were significant (F = 58.69, *p* < 0.001). As shown in Figure 1, in 2019, 2020, and 2021, medical students had the identified the highest with professional behaviors (4.04, 4.04, and 4.02, respectively) (ANOVA: F = 15.69, *p* < 0.001), followed by professional emotion (3.84, 3.86, and 3.92) (ANOVA: F = 111.07, *p* < 0.001), professional expectation (3.69, 3.79, and 3.79) (ANOVA: F = 56.41, *p* < 0.001), and professional cognition (3.61, 3.58, and 3.68) (ANOVA: F = 363.76, *p* < 0.001). PI was the highest among all four dimensions in 2021. Specifically, identity to professional cognition was highest in Q1 in all three years (4.37, 4.22, and 4.19); identity to professional emotion was highest in Q4 in all three years (3.95, 3.97, and 4.00); identity to professional behavior was highest in Q8 in 2019 (4.08) and 2020 (4.06), and Q9 in 2021 (4.11); and identity to professional expectation was highest in Q11 in all three years (4.01, 4.00, and 3.97) (Figure 3).

### Logistic Regression for the Influencing Factors of the Professional Cognition

Table 3 showed the logistic regression results of the three models. In all the three models, medical students living in rural areas were found to have a higher possibility of having their PI scores higher than 3 [aOR and 95% CI of model 1, 2, and 3: 1.14 (1.10, 1.17), 1.22 (1.15, 1.29), and 1.23 (1.12, 1.34), respectively]. Education years of father was a negative influencing factor of medical students' PI scores in the three models, and the aOR and its 95% CI were 0.99 (0.99, 0.99), 0.98 (0.97, 0.99), and 0.98 (0.97, 0.99) per year, respectively. As shown in the three models, ideal profession before college and motivation of major selection could also positively influence medical students' PI scores.

**Table 3:** Factors influenced professional identity of medical students in China by multivariate logistic regression models.

**Factors**	**2019–2021** **(244,040 individuals)**		**2019–2021** **(68,872 individuals)**		**2020 (30,147 individuals** **with variables related to** **COVID-19 pandemic)**	
	***aOR* (95% *CI*)**	***P*-value**	***aOR* (95% *CI*)**	***P*-value**	***aOR* (95% *CI*)**	***P*-value**
**Survey year**						
2019	1.00		1.00			
2020	0.75 (0.70, 0.81)	<0.001	0.88 (0.81, 0.96)	0.003		
2021	0.89 (0.83, 0.96)	0.002	1.15 (1.05, 1.25)	0.002		
**Sex**						
Male	1.00		Excluded		Excluded	
Female	1.35 (1.31, 1.38)	<0.001				
**Family location**						
Urban	1.00		1.00		1.00	
Rural	1.14 (1.10, 1.17)	<0.001	1.22 (1.15, 1.29)	<0.001	1.23 (1.12, 1.34)	<0.001
**Family medical background**						
No	1.00		Excluded		Excluded	
Yes	1.06 (1.03, 1.10)	<0.001				
**Type of high school**						
Key high school	1.00		Excluded		1.00	
Ordinary high school	0.97 (0.94, 0.99)	0.04			1.01 (0.93, 1.10)	0.81
Secondary vocational colleges	0.94 (0.88, 1.00)	0.05			0.42 (0.23, 0.76)	0.004
private school	0.57 (0.43, 0.74)	<0.001				
**Years of education of father**						
0	1.00		1.00		1.00	
Per year	0.99 (0.99, 0.99)	<0.001	0.98 (0.97, 0.99)	<0.001	0.98 (0.97, 0.99)	0.007
**Family region**						
Municipality directly under the Central Government/special administrative region	1.00		1.00		1.00	
Eastern	0.96 (0.91, 1.02)	0.24	1.02 (0.91, 1.14)	0.75	0.96 (0.79, 1.17)	0.69
Central	0.95 (0.89, 1.01)	0.09	1.13 (1.01, 1.27)	0.03	1.11 (0.91, 1.35)	0.30
Western	0.93 (0.87, 0.98)	0.01	1.04 (0.93, 1.16)	0.54	0.97 (0.80, 1.18)	0.74
**College location**						
Wuhan	Excluded		1.00		1.00	
Hubei except Wuhan			1.37 (1.06, 1.79)	0.02	1.97 (1.28, 3.04)	0.002
China except Hubei			1.41 (1.17, 1.70)	<0.001	1.45 (1.10, 1.92)	0.008
**College type**						
Medical school of comprehensive university	1.00		Excluded		Excluded	
Independent medical college	0.93 (0.90, 0.96)	<0.001				
**Ideal profession was related to medicine in high school**						
No	1.00		1.00		1.00	
Yes	1.69 (1.65, 1.74)	<0.001	1.58 (1.49, 1.66)	<0.001	1.46 (1.34, 1.59)	<0.001
**Intrinsic motivation score of major selection**						
≤3	1.00		1.00		1.00	
>3	6.63 (6.44, 6.82)	<0.001	3.51 (3.32, 3.71)	<0.001	3.33 (3.04, 3.64)	<0.001
**Extrinsic motivation score of major selection**						
≤3	1.00		1.00		1.00	
>3	2.16 (2.10, 2.22)	<0.001	1.53 (1.45, 1.62)	<0.001	1.42 (1.30, 1.56)	<0.001
**Positive medical behavior scores**						
≤3			1.00		1.00	
>3			4.44 (4.14, 4.77)	<0.001	5.52 (4.91, 6.21)	<0.001
**Negative medical behavior scores**						
≤3			1.00		1.00	
>3			1.26 (1.14, 1.39)	<0.001	1.38 (1.22, 1.56)	<0.001
**Positive teaching behavior scores**						
≤3			1.00		1.00	
>3			2.47 (2.30, 2.65)	<0.001	2.35 (2.09, 2.64)	<0.001
**Negative teaching behavior scores**						
≤3			1.00		Excluded	
>3			1.17 (1.08, 1.27)	<0.001		
**Personal events score during clinical practice**						
≤3			1.00		Excluded	
>3			0.83 (0.75, 0.92)	<0.001		
**Personal comprehensive quality ranking**						
<10%			1.00		1.00	
11–25%			0.83 (0.76, 0.92)	<0.001	0.83 (0.73, 0.95)	0.007
26–50%			0.74 (0.68, 0.81)	<0.001	0.80 (0.70, 0.91)	0.001
51–75%			0.69 (0.63, 0.76)	<0.001	0.72 (0.63, 0.83)	<0.001
>75%			0.46 (0.41, 0.51)	<0.001	0.47 (0.40, 0.56)	<0.001
**Attention to the COVID-19 pandemic**						
General					1.00	
More					1.93 (1.67, 2.24)	<0.001
Most					2.31 (2.00, 2.68)	<0.001
**Participation of parents or teachers on the front lines of COVID-19 pandemic**						
Neither parent or teacher					1.00	
Only parents					0.72 (0.57, 0.93)	0.01
Only teachers					1.09 (1.00, 1.18)	0.07
Both parents and teachers					1.01 (0.87, 1.17)	0.87
**Hosmer and lemeshow test**	χ^2^ = 51.101, f=8, P < 0.001		χ^2^ = 198.884, f=8, P < 0.001		χ^2^ = 75.450, f=8, P < 0.001	

*aOR, adjusted odds ratio; CI, confident interval; COVID-19, novel coronavirus disease 2019*.

Model 2 and 3 examined the influence of clinical practice on PI scores. Both medical behavior and teaching behavior (regardless of positive or negative) had positive influence on medical students' PI scores. However, Model 2 showed that personal events during clinical practice could negatively influence medical students' PI scores (aOR = 0.83, 95% CI, 0.75, 0.92). Model 2 and 3 also showed that the higher medical students' personal comprehensive quality ranking, the higher their PI scores.

Model 3 further examined the influence of factors related to the COVID-19 pandemic on medical students' PI scores. The attention to the COVID-19 pandemic could positively influence PI score as evidenced by the following: aOR of more attention was 1.93 (95% CI, 1.67, 2.24), and aOR of most attention was 2.31 (95% CI, 2.00, 2.68). However, medical students whose parents participated on the front lines of COVID-19 pandemic had lower PI scores (aOR = 0.72, 95% CI, 0.57, 0.93).

## Discussion

To the best of our knowledge, this is the first comprehensive effort to assess the PI of medical students in China using the data from the three national cross-sectional surveys of CMSS in 2019, 2020, and 2021. We additionally conducted a multivariable logistic regression to find out what factors could influence the PI of medical students in China. The overall PI increased from 2019 (3.80) to 2021 (3.85), and students had the highest identity to professional behaviors and the lowest identity to professional cognition. All the three logistic regression models showed that family located in rural areas and low educational years of father had positive impact on PI. Professional ideal career in high school and the motivation of major selection could also influence the PI. Students' experience during clinical practice impacted the PI in many ways. The more attention students paid to the COVID-19 pandemic, the higher PI they would have. Parents' participation on the front lines of COVID-19 pandemic negatively impacted the PI of medical students.

Our results showed that the overall PI increased from 2019 to 2021. However, the logistic regression results showed that, in all the participants, PI in 2020 (*OR* = 0.75, 95% *CI*, 0.70–0.81) and 2021 (*OR* = 0.89, 95% *CI*, 0.83–0.96) was lower than in 2019. Meanwhile, in those having experienced clinical practice, PI was also lower in 2020 (*OR* = 0.88, 95% *CI*, 0.81–0.96) but higher in 2021 (*OR* = 1.15, 95% *CI*, 1.05–1.25) than in 2019. CMSS in 2020 was conducted in June and July, when medical students had been retracted from offline education and clinical practice for over 6 months due to the COVID-19 pandemic. Due to absence of peer interactions and omission of direct patient care involvement, there was increasing barriers in PIF as medical students struggled with finding their worth in healthcare (13). Study had shown that the lockdown and the school closure could have negative consequences on students, affecting their education, social life, and mental health (14). Nevertheless, online education did ensure that students get uninterrupted learning during the COVID-19 pandemic. Thus, the curriculum and clinical practice could be carried out effectively with the re-opening of universities in China, which might explain the increased PI in students who had clinical practice experiences in the 2021 survey. Additionally, the promotion of medical students' PI may also be related to the important role played by health workers in the COVID-19 pandemic and the timely and effective prevention and control of COVID-19 in China, which imperceptibly improves the professional pride of medical students in China.

Family influence is an important factor of PIF. Our results showed that medical students from rural areas had higher PI than those from cities. Low educational years of fathers had a small but statistically significant positive effect on medical students' PI. Notably, students whose parents participated on the front lines of COVID-19 pandemic had relatively low PI (OR = 0.72, 95% CI, 0.57–0.93). For these students, the fact that their parents were under the high risk of infection and kept away from their families might affect the PIF despite their heroic contributions for fighting against the pandemic. In addition, a qualitative study found that healthcare workers on the front lines might also had their PI affected by factors such as the “impression of exhaustion and fear,” “feeling the unfairness,” “perceiving incompetence in rescue task,” and “unexpected professional benefits.” (15). We also found that students studying outside of Wuhan province had higher PI than those study in Wuhan. The COVID-19 pandemic let medical students and professions discover a “hidden corner” in themselves. Some had managed to overcome these emotional struggles to fulfill their responsibilities, but some had not (16).

During medical education and clinical practice, teachers' role model (regardless of positiveness or negatives) could all positively influence medical students' PI. Despite this, personal events during clinical practice can also negatively influence the PI (OR = 0.83, 95% CI, 0.75–0.92). Meanwhile, students with higher personal comprehensive quality ranking had higher PI. During clinical practice, medical students reported that clinical teachers exert an important influence on their PIF through role modeling, formal and informal teaching, mentoring, assessment, feedback, and interpersonal interactions (17–20). Sternszus et al. explored the PIF from the perspective of clinical teachers and found that participating teachers identified explicit role modeling, engaging in difficult conversations, and providing graded autonomy as ways in which they could influence the identity development of medical students (21). These findings emphasized the importance of teachers' role in medical students' PIF. A previous study also showed that social interactions with others in the clinical practice could help medical students to construct their PI (22). Therefore, experiencing personal events (such as being publicly humiliated or being asked to deal with the personal affairs of others) in the clinical practice had a negative impact on the PI.

Our results also showed that the more attention students paid to the COVID-19 pandemic, the higher PI they would have. During the COVID-19 pandemic, medical students were uncertain about their roles but were eager to contribute (7). The pandemic not only brought pain or death, but also brought attention to medical humanity education, making more medical students realize that the core of medicine is humanity (16). The COVID-19 pandemic has also motivated many high-school students in China to choose medical schools in the National College Entrance Examination (23). Therefore, the pandemic is a crisis and an opportunity, and educators can harness this chance for growth.

### Limitations and Strengths

This study has several limitations. First, the first 2 years' survey were anonymous. Hence, we could not track the change of personal PI by a cohort study. Second, these surveys were conducted in China, and due to the differences in disease control policies, the results have limited generalizability. However, our findings may be useful in undergraduate medical education settings, because the COVID-19 pandemic and its consequences on medical education have been global. Third, the three surveys were all conducted at a time when the pandemic situation was relatively stable (the peak of the COVID-19 pandemic in China was from January to March in 2020 (24)), and the results of our study may be overestimated or underestimated due to the difference of PI between peak and off peak of the pandemic. Nevertheless, our study also had strengths. To the best of our knowledge, this is the first comprehensive effort to assess the PI of medical students in China using the data from the three national cross-sectional surveys of CMSS in 2019, 2020, and 2021. We quantified medical students' PI and additionally conducted multivariable logistic regression to find out what factors influenced the PI of medical students in China.

## Conclusions

The PI of medical students in China increased during the COVID-19 pandemic compared with before. Timely and effective prevention and control of COVID-19 could improve PI of medical students. Sociodemographic characteristics, medical education, and clinical practice experience can influence medical students' PI. The association of the pandemic and medical students' PI was complex. The PI was positively influenced by students' attention to the pandemic, but negatively influenced by participation of parents on the front lines. A well-formed PI during medical education and clinical practice is of vital importance for medical students to quickly adapt to professional status and to better deal with practical challenges. Concerted efforts should be made by the education sector, health sector, and the society to improve the PI of medical students and health care workers.

## Data Availability Statement

The raw data supporting the conclusions of this article will be made available by the authors, without undue reservation.

## Ethics Statement

The studies involving human participants were reviewed and approved by Institutional Review Boards at Peking University. The patients/participants provided their written informed consent to participate in this study.

## Author Contributions

JL and CY conceived and designed the study. CY, WW, and AX did the questionnaire design, colleges connection, data collection, and cleaning. QL did a literature search, analysis and interpretation, compiled tables and figures, and drafted the manuscript. JL, CY, QL, WW, and AX revised the manuscript. All authors contributed to the article and approved the submitted version.

## Funding

This study was funded by the Major Project of the National Social Science Foundation of China (AIA210011). The funders had no role in study design, data collection and analysis, decision to publish, or preparation of the paper.

## Conflict of Interest

The authors declare that the research was conducted in the absence of any commercial or financial relationships that could be construed as a potential conflict of interest.

## Publisher's Note

All claims expressed in this article are solely those of the authors and do not necessarily represent those of their affiliated organizations, or those of the publisher, the editors and the reviewers. Any product that may be evaluated in this article, or claim that may be made by its manufacturer, is not guaranteed or endorsed by the publisher.
