# Supplementary material for: Professional Identity of 0.24 Million Medical Students in China Before and During the COVID-19 Pandemic: Three Waves of National Cross-Sectional Studies
Source: Front Public Health. 2022 Mar 25;10:868914. doi: 10.3389/fpubh.2022.868914 (PMC8989960; doi:10.3389/fpubh.2022.868914)
Supplement: Supplementary file 1 [file Table_1.DOCX]

Supplementary Table 1. Indicators of motivation of major selection, doctors’ role model, clinical practice events, and professional identity.

| **Primary indicators** | **Secondary indicators** | **Tertiary indicators** |
| --- | --- | --- |
| Motivation of major selection | Intrinsic motivation | The scores of disciplines related to this major were good in senior high school |
|  |  | Having a strong interest in medicine |
|  |  | I think being a doctor is respected |
|  |  | Being confident of success in this field |
|  |  | Doctors are a sacred and noble cause of saving the lives and healing the wounded |
|  | Extrinsic motivation | Doctors are stable professions |
|  |  | My parents and relatives encouraged / asked me to choose this major |
|  |  | Convenience of receiving healthcare for individuals, family and friends in the future |
|  |  | The future salary of clinical medicine is high |
| Doctors' role model | Positive medical behaviors | Medical workers love their medical work |
|  |  | Medical workers are very responsible in medical work |
|  | Negative medical behaviors | Medical workers are reluctant to communicate with patients |
|  | Positive teaching behaviors | Medical workers pay attention to the role model for medical students |
|  |  | Medical workers pay attention to the influence of their words and deeds on students in teaching |
|  | Negative teaching behaviors | Medical workers did not focus on teaching and did not pay attention to teaching |
| Clinical practice events | Medical events | Experience doctor-patient conflict |
|  |  | Experience medical errors |
|  | Personal events | Being publicly humiliated |
|  |  | Being treated unfairly |
|  |  | Being made difficult in the process of doing things |
|  |  | Being asked to do other people's personal things |
| Professional Identity | Professional cognition | I know my responsibilities as a doctor |
|  |  | Doctor is one of the best professions |
|  |  | Being a doctor is not just a means of making a living |
|  | Professional emotion | Medical education made me more determined to become a doctor |
|  |  | When communicating with students of other majors, I am very proud that I am a medical student |
|  |  | Doctor is my ideal profession |
|  |  | Being a doctor is very respected |
|  | Professional behavior | I will actively participate in relevant academic and practical activities conducive to my medical career |
|  |  | I want to be a doctor after graduation |
|  |  | I will pay attention to the latest research progress in medical field |
|  | Professional expectation | I am confident of becoming an excellent doctor |
|  |  | I am optimistic about the development prospect of the medical and health industry |
